# Alternative Fillers in Asphalt Concrete Mixtures: Laboratory Investigation and Machine Learning Modeling towards Mechanical Performance Prediction

**DOI:** 10.3390/ma16020807

**Published:** 2023-01-13

**Authors:** Nitin Tiwari, Fabio Rondinella, Neelima Satyam, Nicola Baldo

**Affiliations:** 1 Lyles School of Civil Engineering, Purdue University, West Lafayette, IN 47907, USA; 2 Department of Civil Engineering, Indian Institute of Technology Indore, Indore 452020, India; 3 Polytechnic Department of Engineering and Architecture (DPIA), University of Udine, Via del Cotonificio 114, 33100 Udine, Italy

**Keywords:** asphalt mixtures, recycling, waste materials, alternative fillers, artificial intelligence, machine learning, decision tree, CatBoost

## Abstract

In recent years, due to the reduction in available natural resources, the attention of many researchers has been focused on the reuse of recycled materials and industrial waste in common engineering applications. This paper discusses the feasibility of using seven different materials as alternative fillers instead of ordinary Portland cement (OPC) in road pavement base layers: namely rice husk ash (RHA), brick dust (BD), marble dust (MD), stone dust (SD), fly ash (FA), limestone dust (LD), and silica fume (SF). To exclusively evaluate the effect that selected fillers had on the mechanical performance of asphalt mixtures, we carried out Marshall, indirect tensile strength, moisture susceptibility, and Cantabro abrasion loss tests on specimens in which only the filler type and its percentage varied while keeping constant all the remaining design parameters. Experimental findings showed that all mixtures, except those prepared with 4% RHA or MD, met the requirements of Indian standards with respect to air voids, Marshall stability and quotient. LD and SF mixtures provided slightly better mechanical strength and durability than OPC ones, proving they can be successfully recycled as filler in asphalt mixtures. Furthermore, a Machine Learning methodology based on laboratory results was developed. A decision tree Categorical Boosting approach allowed the main mechanical properties of the investigated mixtures to be predicted on the basis of the main compositional variables, with a mean Pearson correlation and a mean coefficient of determination equal to 0.9724 and 0.9374, respectively.

## 1. Introduction

An in-service flexible road pavement is usually affected by deteriorating factors such as traffic, water, and aging [1]. For this reason, it is crucial that the designed asphalt mixture (AM) has suitable mechanical performance. AMs mostly consist of coarse aggregates and bitumen, but also a certain percentage of filler must be added and balanced. A proper amount of filler helps both in reducing the mixture porosity and in hardening the bitumen, improving the overall performance in terms of rutting and shoving response [2,3,4,5,6,7]. Conversely, an excessive amount of filler decreases the aggregate content, thus leading to a strength reduction [8]. Over the years, researchers have investigated the effects that different mineral fillers produced in the mixtures they were used for [9,10,11,12,13], and it was determined that cement produced the best improvements in AMs’ mechanical performance [14]. However, increasing concern about environmental sustainability has highlighted the high CO_2_ emissions related to cement manufacturing. For this reason, a valuable alternative that is both environment-friendly and mechanically sound should be found [15]. A successful substitution requires the complete characterization of the recycled material or industrial waste to be reused. The most critical parameters that could affect the overall performance of the AM must be determined, namely: filler particle shape and size, specific gravity, surface area, mineralogical composition, and the presence of potentially harmful fine material [16,17]. In this framework, several waste materials have already been analyzed as potential alternative fillers, such as: copper slag powder [18], bauxite residue [19], empty palm fruit bunch ash [20], and coffee husk ash [21].

Recently, the mechanical behavior of asphalt mixtures prepared with marble dust has proven to be technically sound and preferable to what can be achieved using fillers obtained from steel slag or granite [22]. Low percentages of SiO_2_ coupled with high percentages of CaO result in a higher water resistance and reduced moisture-induced damage [23]. The possibility of achieving similar or even slightly better performance by replacing traditional fillers with waste materials and the simultaneous reduction in waste disposal problems both encouraged the present study. The study focused on the investigation of AMs prepared with several materials as alternative fillers instead of ordinary Portland cement (OPC), which was used as a reference. The mechanical characterization of rice husk ash (RHA), brick dust (BD), marble dust (MD), stone dust (SD), fly ash (FA), limestone dust (LD), and silica fume (SF) was carried out, together with an in-depth comparative evaluation. Furthermore, Marshall, indirect tensile strength (ITS), moisture susceptibility and Cantabro abrasion loss (CL) tests were performed on AMs prepared with these fillers to evaluate their influence on the corresponding mixture’s behavior. Four different filler contents were considered, ranging from 4% to 8.5% by volume of the mixture, with a step-size of 1.5%.

The resulting experimental data were processed by a machine learning (ML) model based on a modern Categorical Boosting (or CatBoost) approach. In the last few decades, support-vector machines, artificial neural networks, and decision-tree-based models are gaining wide approval in the research community since they are able to predict the mechanical behavior of AMs in a faster and more accurate way than empirical or statistical techniques [24,25]. Although they neglect the physical nature of the investigated problem, they can provide a reliable alternative to the advanced constitutive equations related to the mechanics of materials [26,27,28,29,30]. Several successful ML approaches have been established over the years [31,32,33], thus allowing the mechanical behavior of AMs to be predicted in terms of fatigue life [34], tensile strength [35], and international roughness index [36,37]. For these reasons, the second goal of this study was to develop a ML CatBoost approach capable of predicting the mechanical response of AMs based on a few compositional variables. Despite the fact that relatively few data were obtained from the laboratory investigation, an innovative data augmentation technique was implemented to expand the size of the starting dataset, thus allowing the model to be properly trained.

The core aspect of the present study can be found in the recycling of waste materials as alternative fillers in asphalt mixtures with the simultaneous reduction in waste disposal problems. Furthermore, a direct comparison of the performance shown by mixtures prepared with eight alternative fillers, along with the use of up-to-date machine learning techniques to predict the mixtures’ mechanical behavior, represent the innovative aspect of this research. The result was an integrated approach that, to determine and predict the mechanical response of AMs prepared with selected fillers, involved both an in-depth experimental investigation and the development of a proper ML model.

## 2. Materials and Methods

### 2.1. Aggregate, Bitumen and Fillers

Sharp-edged aggregates from crushed quartz were used in this investigation. They were supplied by Safew Tech System (Indore, India), and several laboratory tests were carried out to fully characterize them. A detailed overview of the parameters determined, the procedures followed, the results obtained, and the limits set by the Indian Ministry of Road Transport and Highways (MoRTH) has been provided in Table 1.

**Figure 1:**
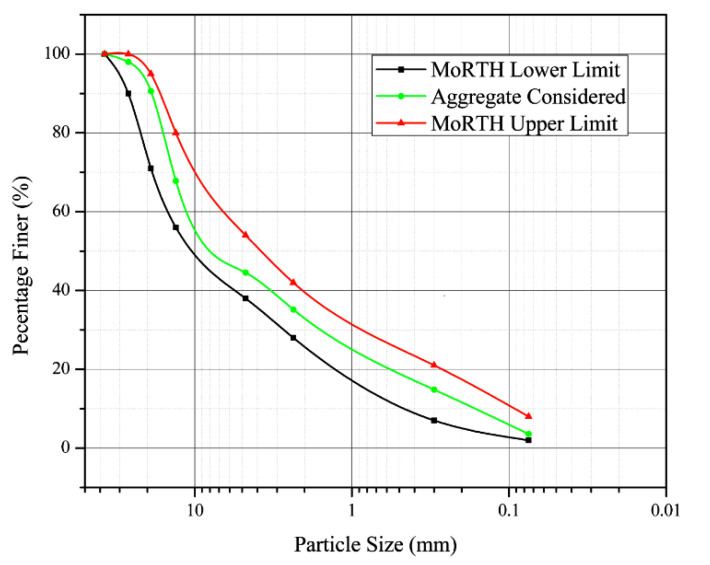
Aggregate grading curve with MoRTH upper and lower limits.

The aggregate grading curve was then compared with the reference envelope specified by the MoRTH about flexible road pavements. The material was consequently classified as grade II, and the graphical representation of this comparison has been provided in Figure 1.

Tiki Tar Industries is one of India’s largest private sector bitumen companies, and it supplied the conventional VG–30 bitumen used in this investigation. Also in this case, a detailed overview of the parameters determined, the procedures followed, the results obtained, and the limits set by the MoRTH has been provided in Table 2.

The filler used as a reference is ordinary Portland cement (OPC), since this is the material most widely used to prepare asphalt mixtures for base course layers in heavy-load road pavements in India. Seven additional mineral fillers were analyzed, and a summary of the comparative analysis is provided in Table 3. Both physical and chemical properties were investigated in terms of specific gravity, methylene blue value (MBV), German filler [38], fineness modulus (FM), surface area, pH, main oxide contents, and particle shape.

**Figure 2:**
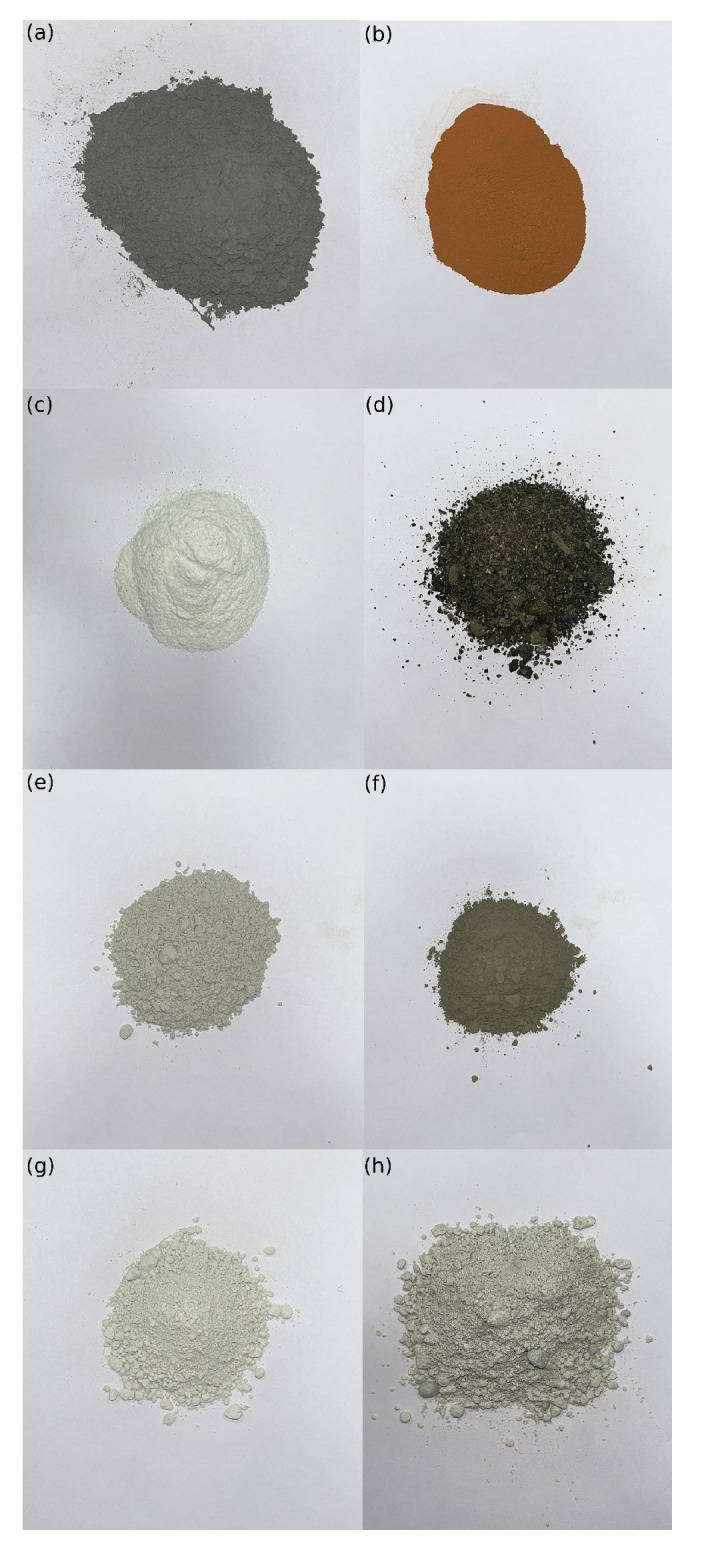
RHA (**a**), BD (**b**), MD (**c**), SD (**d**), FA (**e**), OPC (**f**), LD (**g**), and SF (**h**) filler materials.

To evaluate exclusively the physical–mechanical impact that the selected fillers had on reference mixtures, some volumetric variables were kept constant. Aggregate type, grading curve, and bitumen type remained unchanged. The optimum bitumen content for each asphalt mixture was evaluated. Conversely, eight selected fillers and four different filler contents (4.0, 5.5, 7.0, and 8.5% by percentage volume of the mixture) were considered, resulting in a total of 32 analyzed mixtures. It should be considered that the cumulative percent passing 0.6, 0.3, and 0.075 mm sieves had to be equal to 100%, 95–100%, and 85–100% by weight of total aggregate, respectively. Furthermore, the grading percentage of the 0.075 fraction ranged between 2–8% in accordance with MoRTH standards. The particle shape of each filler is shown in Table 3. RHA, BD, MD, SD, FA, OPC, LD, SF, (Figure 2) and the aggregates were individually preheated to 150 °C. Afterwards, the required amount of aggregate and filler was mixed at 170 °C in a planetary mixer at the speed of 140 ± 5 rpm. The respective amounts of bitumen were then added according to MoRTH standards to prepare the mixtures. Volumetric properties of AMs significantly impact on mechanical strength and long-term durability of road pavements. For this reason, the percentage of air voids (AV), voids in the mineral aggregate (VMA), and voids filled with bitumen (VFB) were measured in accordance with MoRTH standards. To prepare the specimens for the following experimental investigation, the Marshall method was followed. Although this technique has proven to lead to volumetric properties slightly different from those observed in road pavements [39], it is still extensively used in road laboratories [8,40,41,42,43] and is preferred to the SUPERPAVE method [44] because of its simplicity and cost-effectiveness. Before the Marshall tests, each specimen (67 ± 3 mm in height and 102 mm in diameter) was compacted by means of 75 Marshall hammer blows on each side of the specimen. Afterwards, it was air-dried for 4 h and finally held for 35 ± 5 min in a water bath at 60 ± 1 °C. A 100 kN load cell was used along with a 50 mm linear variable displacement transducer to obtain the maximum value of load resistance and the corresponding deformation for a strain rate equal to 50.4 mm/min. The results obtained from this laboratory investigation are hereafter referred to as Marshall stability (MS), flow (MF), and quotient (MQ) in accordance with the ASTM “D 6927 Standard Test Method for Marshall Stability and Flow of Bituminous Mixtures” [45].

MQ is defined as the ratio between MS and MF, and it has been empirically correlated with asphalt concrete rutting performance [46]. The higher the MQ value, the better the resistance to traffic-induced creep deformation shown by the asphalt mixture. Furthermore, ITS tests were carried out for all the mixtures according to the ASTM “D 6931 Standard Test Method for Indirect Tensile Strength (ITS) of Asphalt Mixtures” [47]. Specimens were prepared according to the Marshall protocol, and tensile resistance at 25 °C was determined by applying a constant loading rate equal to 50.8 mm/min on specimen diameters in compression. A modified Lottman test was carried out to evaluate water sensitivity. ITS values were determined before and after moisture conditioning (in accordance with the AASHTO “T 283 Resistance of Compacted Asphalt Mixtures to Moisture-Induced Damage”), and the ratio between them expressed the indirect tensile strength ratio (ITSR). Specimens measuring 150 mm in diameter and 63.5 in thickness were compacted by means of the Marshall hammer targeting an AV content of 7 ± 0.5%. After 24 ± 3 h storing at 25 °C, specimens were saturated at absolute vacuum pressure of 50 kPa for 10 min and left soaking for an additional 10 min in a vacuum container targeting a saturation degree of 75 ± 5%. Specimens were wrapped in a thin plastic film and sealed in a plastic bag containing 10 ± 5 mL of water. Sealed specimens were frozen at –18 ± 3 °C for 24 h. The plastic bag was then removed, and specimens were placed in a water bath at 25 ± 5 °C for 24 h. ITS tests were carried out on conditioned specimens according to ASTM D 6931 [47].

Finally, to account for mixture degradation behavior, a Cantabro abrasion loss test was carried out according to the TxDOT designation Tex-245-F [48]. Specimens measuring 101 ± 1.5 mm in diameter and 50.8 ± 1.5 mm in thickness were compacted by means of the Marshall hammer with 50 blows for each side. The initial weight of the test specimen at 25 °C ($m_{i}$) was determined. After 24 h, the specimen was placed in a Los Angeles testing machine without the abrasive charges. The test was carried out at the speed of 32 rpm for 300 revolutions. Loose material broken off the test specimen was discarded, and then the final weight of the test specimen ($m_{f}$) was determined. Cantabro loss was calculated according to Equation (1):

(1) $$CL=\frac{m_{i}-m_{f}}{m_{i}}\times 100$$

### 2.2. CatBoost Model

Data from the laboratory investigation were analyzed by means of a modern ML technique called CatBoost. This is an innovative algorithm that stems from the decision- tree-based Gradient Boosting, but solves the main drawbacks commonly referred to as prediction shift and target leakage [49]. Combining the implementation of decision tables [50] and Ordered Boosting [49], CatBoost defines a more efficient and balanced tree architecture that outperforms current decision-tree-based algorithms [51] such as XGBoost [52] and LightGBM [53]. Furthermore, CatBoost allows datasets containing categorical variables to be automatically processed and analyzed. For a deeper insight into CatBoost algorithm functioning, a formal description can be found in the work of Prokhorenkova et al. [49]. To optimize the performance of the CatBoost model, its hyperparameters need to be carefully fine-tuned. An extensive grid search (Table 4) was implemented to identify the best values in terms of number of trees to be built (number of iterations), maximum depth of such trees (max depth), and gradient step size (learning rate). To avoid overfitting phenomena, a k-fold cross-validation procedure [54] and an overfitting detector were implemented in addition to the maximum tree depth condition [55]. Since 80% of the dataset was used for model training and validation, the k-value was set equal to 4 so that each fold included the same number of observations. The remaining 20% was used for the testing phase. The overfitting detector was set equal to its default value of 20 according to the relevant literature [56]. The best model was determined by minimizing the value of the loss function. The most suitable function to accomplish this task was identified in *MultiRMSE* because the goal was the simultaneous prediction of 5 parameters (MS, MQ, ITS, ITSR, and CL) on the basis of 3 inputs, namely: a categorical variable identifying the filler type (CV), the filler content (Fc), and the air void percentage (AV).

Let D be the number of output variables, N the number of observations included in the test vector, $y_{T_{i}}$ the *i*-th target, and $y_{P_{i}}$ the *i*-th prediction. The analytical expression of *MultiRMSE* is then determined as follows:

(2) $$MultiRMSE=\sqrt{\frac{1}{N}\overset{N}{\underset{i=1}{\sum }}\overset{D}{\underset{d=1}{\sum }}{\left(y_{T_{i,d}}-y_{P_{i,d}}\right)}^{2}}$$

To accurately measure the reliability of the predictions, multiple performance metrics were implemented. Their definitions and analytical expressions are provided in Table 5. The meanings of the terms were kept consistent with the above; µ and σ refer to the mean-value and standard deviation of each variable, respectively. The full methodology was implemented in Python 3.8.5.

### 2.3. Data Augmentation

**Figure 3:**
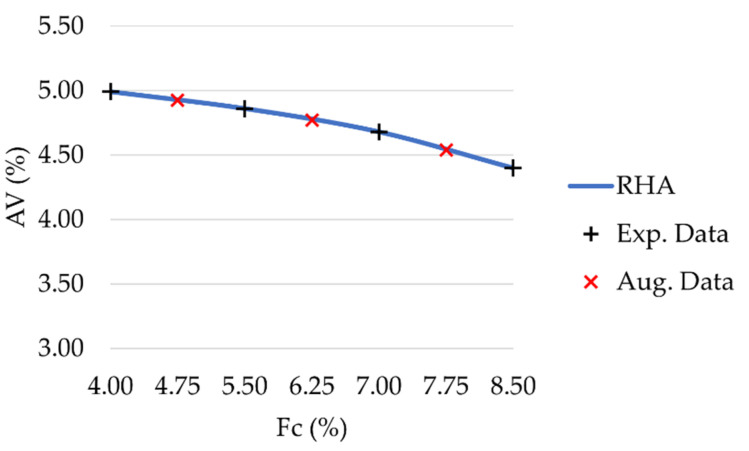
Experimental data (black plus sign marker) and augmented data (red cross marker) for RHA mixtures.

The performance of a machine-learning-based model is highly dependent on training data’s quality, quantity and meaning. The small size of the datasets is one of the main challenges in model development, since collecting a considerable amount of data often requires a very long time and very high costs. Data augmentation techniques artificially increase the available amount of data by generating new synthetic data based on the collected experimental observations. However, a fundamental condition must be satisfied: the augmented data must keep the original meaning of the experimental data without altering it [57]. This request is easy to satisfy for image classification problems, since cropping, zooming, or rotating an image does not modify its meaning. However, this condition becomes trickier in time-series predictions or input-target fitting problems. In the field of pavement engineering, small-size datasets frequently occur since most laboratory investigations are expensive and time-consuming. For this reason, mechanical behavior analyses are often limited to a small number of specimens and few variations of a given asphalt concrete mixture. In this study, to estimate unknown values based on known data, the modified Akima (Makima) cubic Hermite interpolation technique was implemented [58]. This improves on the original Akima algorithm [59], and consists in producing piecewise polynomials with continuous first-order derivatives by performing a cubic interpolation. In this way, even highly nonlinear problems with oscillatory data can be considered, without the excessive local undulations of third degree polynomial or cubic spline interpolation. In accordance with Oh et al. [57], original data were not outnumbered by augmented data. It was implemented a Makima algorithm aimed at determining the feature value between two successively investigated filler contents, resulting in 3 synthetic observations for each curve. This was performed for each of the eight mineral fillers considered and provided 24 additional data points for each feature. The dataset size was nearly doubled, increasing from 32 to 56 observations, and an exemplificative diagram related to the air voids of mixtures prepared with RHA was produced (Figure 3).

## 3. Results and Discussion

### 3.1. Laboratory Results

**Figure 4:**
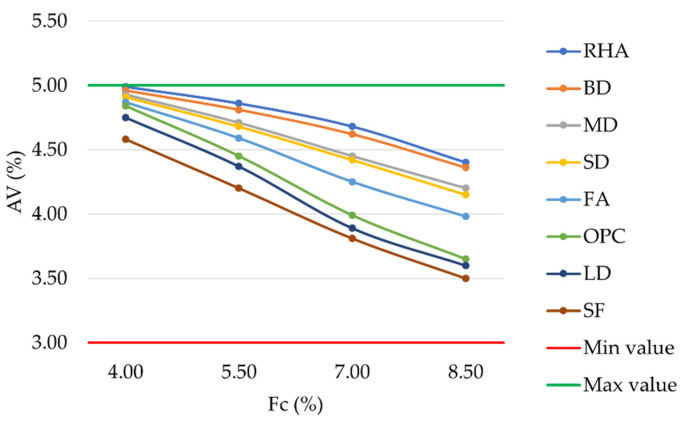
Air voids vs. filler content for mixtures prepared with conventional and waste fillers.

**Figure 5:**
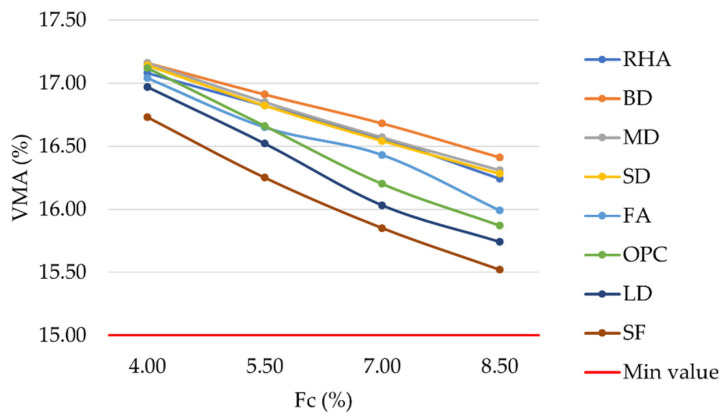
Voids in the mineral aggregate vs. filler content for mixtures prepared with conventional and waste fillers.

**Figure 6:**
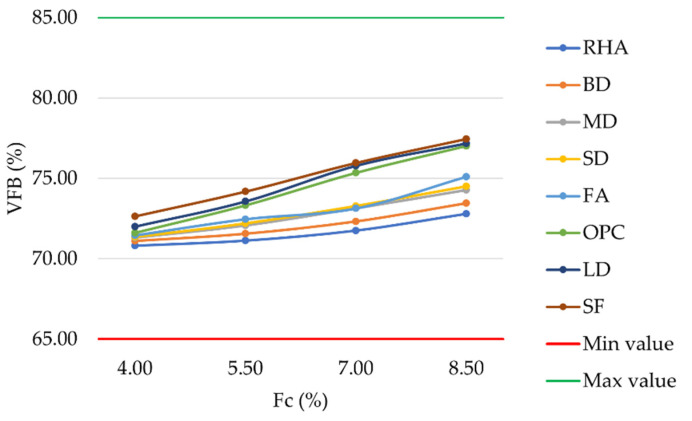
Voids filled with bitumen vs. filler content for mixtures prepared with conventional and waste fillers.

**Figure 7:**
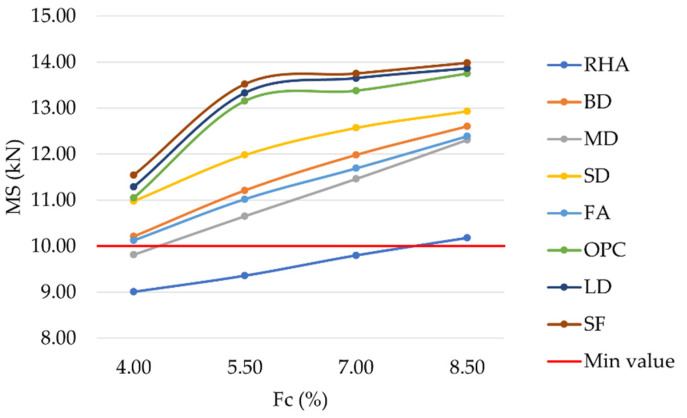
Marshall stability vs. bitumen content for mixtures prepared with conventional and waste fillers.

**Figure 8:**
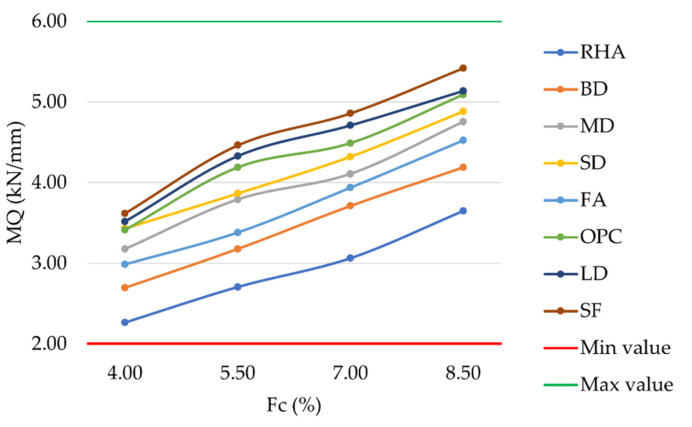
Marshall quotient vs. filler content for mixtures prepared with conventional and waste fillers.

**Figure 9:**
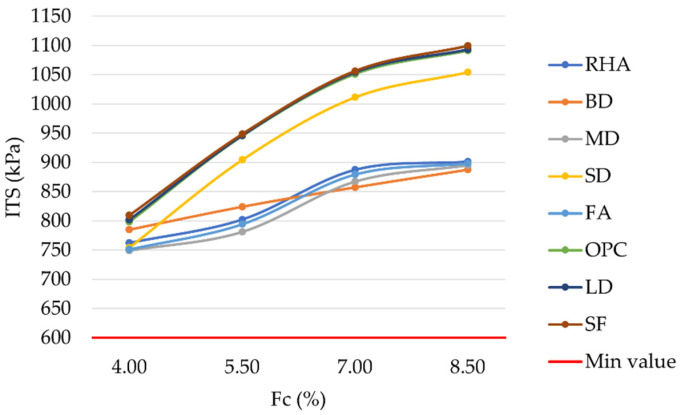
Indirect tensile strength vs. filler content for mixtures prepared with conventional and waste fillers.

**Figure 10:**
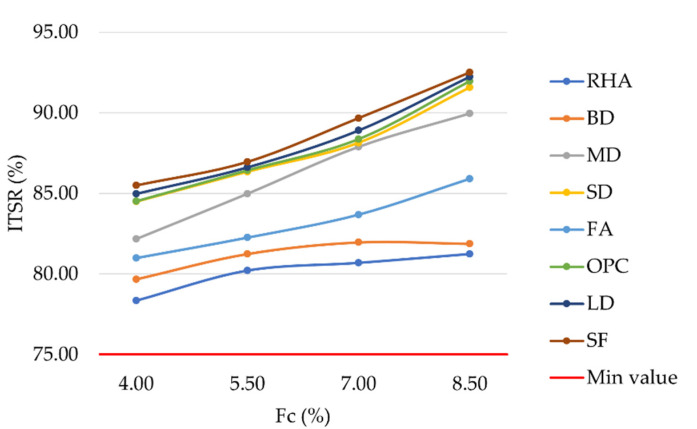
Indirect tensile strength ratio vs. filler content for mixtures prepared with conventional and waste fillers.

**Figure 11:**
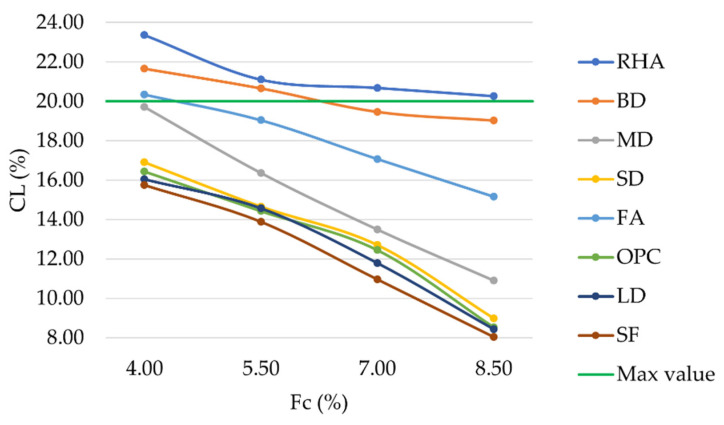
Cantabro loss vs. filler content for mixtures prepared with conventional and waste fillers.

The results of the laboratory investigation are presented in Figure 4, Figure 5, Figure 6, Figure 7, Figure 8, Figure 9, Figure 10 and Figure 11. The trend in the air void percentage as the filler content changes can be observed in Figure 4 for asphalt mixtures prepared with conventional and waste fillers. As expected, an increase in filler content led to a corresponding decrease in air voids. All mixture types met the requirements of MoRTH standards, with AV values within the range of 3%–5%. However, for the same filler contents, LD and SF mixtures showed a lower air void content than OPC mixtures, with differences ranging from 0.05% to 0.26%.

VMA is defined as the volume of intergranular void space between the compacted mixture aggregates, including air voids and unabsorbed bitumen volume. As can be seen in Figure 5, LD and SF mixtures show lower VMA values than OPC mixtures, with differences ranging from 0.13% to 0.41%.

Considering a filler content equal to 8.5%, mixtures prepared with BD, MD, SD, RHA, FA, OPC, LD, and SF showed VMA values equal to 16.41%, 16.31%, 16.28%, 16.24%, 15.99%, 15.87%, 15.74%, and 15.52%, respectively.

The percentage of VMA filled with bitumen defines VFB. It can be noticed that an increase in filler content led to a corresponding increase in the percentage of voids filled with bitumen. In accordance with the previously described AV and VMA values, the substitution of OPC with LD or SF slightly increased VFB in the corresponding mixtures (Figure 6).

Differences ranged from 0.15% to 1.02%. However, all mixture types met the requirements of MoRTH standards, with VFB values within the range of 65%–85% [60]. Considering a filler content equal to 8.5%, mixtures prepared with FA, SD, MD, BD, and RHA showed VFB values lower than OPC mixtures and equal to 75.10%, 74.49%, 74.27%, 73.45%, and 72.80%, respectively. Because of their reduced bleeding possibility, mixtures prepared with these five mineral fillers could be preferred in hot climate regions [61].

The mechanical behavior of the investigated mixtures has been empirically described by means of Marshall parameters. MS is graphically represented in Figure 7. As expected, an increase in filler percentage led to an increase in MS for each bitumen percentage [62,63,64]. Except for the mixtures prepared with 4%, 5.5%, and 7% RHA and 4% MD (9.01 kN, 9.36 kN, 9.80 kN, and 9.81 kN, respectively), all other mixtures showed MS values above the acceptance threshold of 10 kN established by the Indian regulations. The highest MS was reached for the highest filler content with values equal to 10.18 kN, 12.31 kN, 12.39 kN, 12.60 kN, 12.93 kN, 13.74 kN, 13.86 kN, and 13.98 kN for mixtures prepared with RHA, MD, FA, BD, SD, OPC, LD, and SF, respectively. LD and SF mixtures showed similar behavior to OPC, demonstrating that both materials could effectively replace OPC in asphalt concrete mixtures. Finally, for filler contents of 5.5%, 7.0%, and 8.5%, mixtures prepared with these three fillers also satisfied the acceptance requirement of 13 kN required by MoRTH standards for mixtures prepared with modified bitumen.

The laboratory investigation of MF produced results in the range of 2 mm–4 mm, as required by MoRTH standards. As expected, the lowest MF values occurred at the highest filler contents. By identifying higher stability and lower flow values, the more resistant mixtures in terms of permanent deformation at high temperatures can be empirically determined. For each mixture analyzed, the trend in MQ can be observed in Figure 8. An increase in filler content led to a corresponding increase in the MQ. The substitution of OPC with LD or SF slightly increased VFB in the corresponding mixtures, with differences ranging from 0.04% to 0.37%. However, the results confirmed the achievement of MoRTH acceptance requirements (2 kN/mm–6 kN/mm) for all the investigated mixtures. With respect to SD, MD, FA, BD, and RHA mixtures, MQ values were lower than those obtained with OPC.

In order to understand the tensile resistance behavior of the investigated asphalt concrete mixtures, the trend in the ITS as the filler content changed can be observed in Figure 9. As can be observed, an increase in filler content led to a corresponding increase in ITS, with the highest values corresponding to a filler content equal to 8.5% and leading to higher resistance against fatigue cracking. The substitution of OPC with LD or SF left unaltered the ITS response, slightly increasing the ITS values in the corresponding mixtures. Differences ranged from 1 kPa to 11 kPa, demonstrating that LD and SF can suitably replace OPC in asphalt mixtures. MoRTH standards require an ITS above 600 kPa [60]: this condition was verified for all the investigated mixtures, with peak ITS values even higher than 1 MPa.

The laboratory investigation of ITSR (Figure 10) produced results higher than 75%, as prescribed by MoRTH standards. A higher filler content resulted in slightly higher resistance to water damage, with LD and SF mixtures showing higher ITSR values than OPC. Differences ranged from 0.19% to 1.30%. Due to the antistripping properties of portlandite and calcite minerals in SF, these mixtures showed the highest ITSR values. Similarly, due to the calcite mineral present in LD, bitumen filler adhesion was improved, resulting in high ITSR values. SD mixtures showed similar resistance to water damage as OPC ones; whereas, MD, FA, BD, and RHA had lower ITSR values. Fine clays contained in BD reduced the resistance to water damage of the corresponding mixtures. Similar results were also observed by Kuity et al. and by Arabani et al. [65,66]. Finally, the high porosity of RHA resulted in the lowest ITSR values observed.

With respect to Cantabro abrasion loss, it can be observed that higher filler contents always resulted in lower CL values (Figure 11). LD and SF mixtures performed even better than those prepared with conventional OPC. Considering a filler content equal to 8.5%, mixtures prepared with RHA, BD, FA, MD, SD, OPC, LD, and SF showed CL values equal to 20.25%, 19.01%, 15.15%, 10.89%, 8.97%, 8.52%, 8.42%, and 8.04%, respectively. In accordance with Figure 4, lower values for air voids resulted in a lower occurrence of raveling phenomena.

For all mixtures and for all laboratory parameters analyzed, the standard deviations observed ranged between 8% and 12% of the mean values.

### 3.2. CatBoost Modeling Results

The statistical description of the involved modeling variables is provided in Table 6. As previously described, eight selected filler types and four different filler percentages were analyzed having fixed both the bitumen type and content, and the aggregate grading curve. This resulted in 32 original observations for each variable that, combined with the 24 data points augmented by means of Makima interpolation, determined a comprehensive dataset made up of 56 observations for each feature.

**Figure 12:**
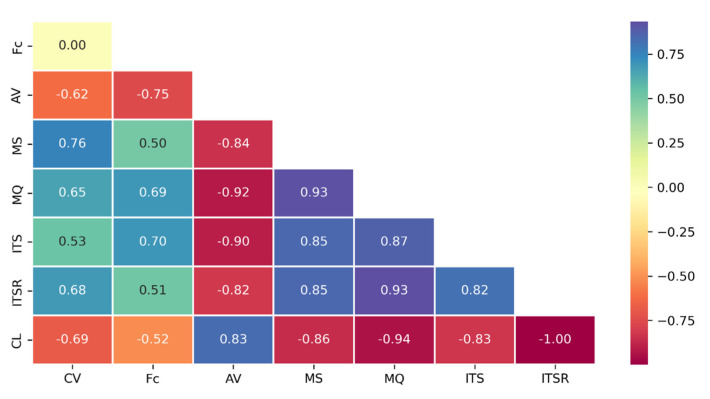
Pearson correlation matrix.

A Pearson correlation matrix [67] was used to preliminarily determine the strength of correlations among the variables considered by the model (Figure 12). Each matrix element is described by a sign and an absolute value. The former stands for direct (+) or inverse (−) proportionality between the two variables under consideration. The latter specifically reports the correlation strength and ranges between 0 and 1. A value of 0 means there is no correlation; the value of one variable cannot be determined based on the value of the other one. Conversely, a value of 1 means there is a perfect correlation; the value of one variable can be exactly determined based on the value of the other one. By way of example, a strong positive correlation was identified between CV and MS [r=+0.76,  n=56,  p<0.0005] and between Fc and ITS [r=+0.70,  n=56,  p<0.0005]. Conversely, a strong negative correlation was identified between AV and MQ [r=−0.92,  n=56,  p<0.0005].

To improve the efficiency of model training [68], all experimental observations of each variable were normalized according to Equation (3). All data were scaled to the same range [0, +1], so that the minimum and the maximum values of each variable corresponded to the lower and the upper bounds, respectively.

(3) $$x_{norm}=\frac{x-x_{min}}{x_{max}-x_{min}}$$

**Figure 13:**
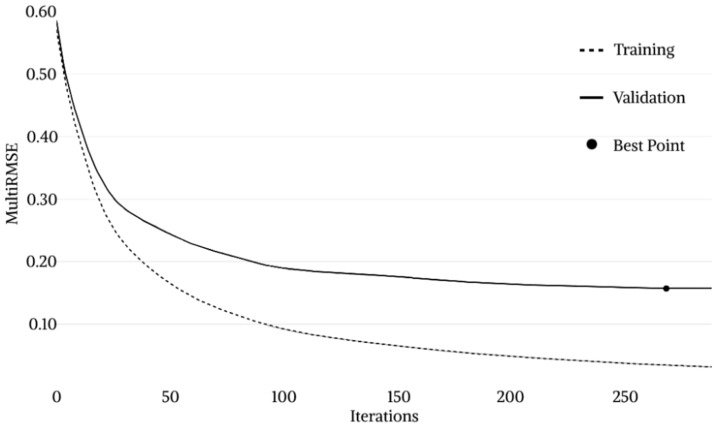
CatBoost model training process.

The CatBoost model training process is represented in Figure 13. In the beginning, both training and validation loss returned high values of about 0.60. As the iterations proceeded, a rapid decrease in loss functions was observed with values settling at around 0.20 after about 50 and 100 iterations for training and validation loss, respectively. The *MultiRMSE* value gradually decreased until the Best Point was reached, identified at iteration 268 by a validation loss value of 0.1569. After this point, a significant decrease in the validation *MultiRMSE* is no longer appreciable. Therefore, the training process was stopped by the overfitting detector after 20 additional iterations. The identification of the best point allows the best hyperparameters configuration to be fixed and to proceed to the successive testing phase.

After the denormalization of the processed variables, the testing phase began. The reliability of model predictions was described by means of six different evaluation metrics, namely MAE, MAPE, MSE, RMSE, R, and R^2^ (Table 7). For each of the five target variables, the mean absolute percentage error was always lower than 5%, with Pearson correlation coefficients higher than 0.95 and coefficients of determination higher than 0.88.

**Figure 14:**
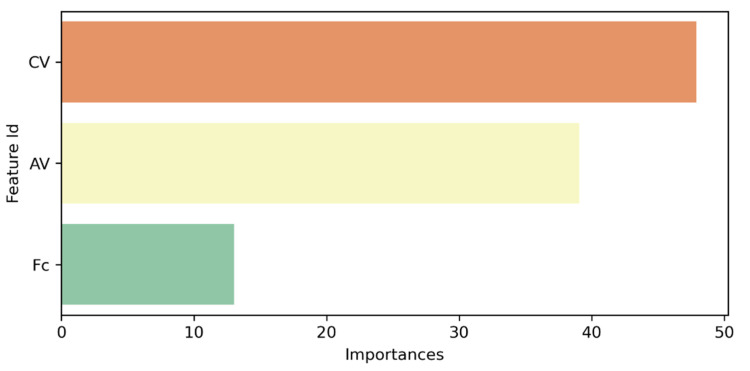
Feature importance.

The influence each variable had on model predictions was determined by means of a sensitivity analysis implemented in Python 3.8.5 (Figure 14). The algorithm allows the importance of each input feature to be determined and normalizes them in order to reach a total of 100%. The more important a given feature is, the more significant will be the average change in predictions if that feature is changed. It can be noticed that the categorical variable has the greatest importance (47.89 %), followed by air voids content (39.07 %) and filler content (13.04 %).

**Figure 15:**
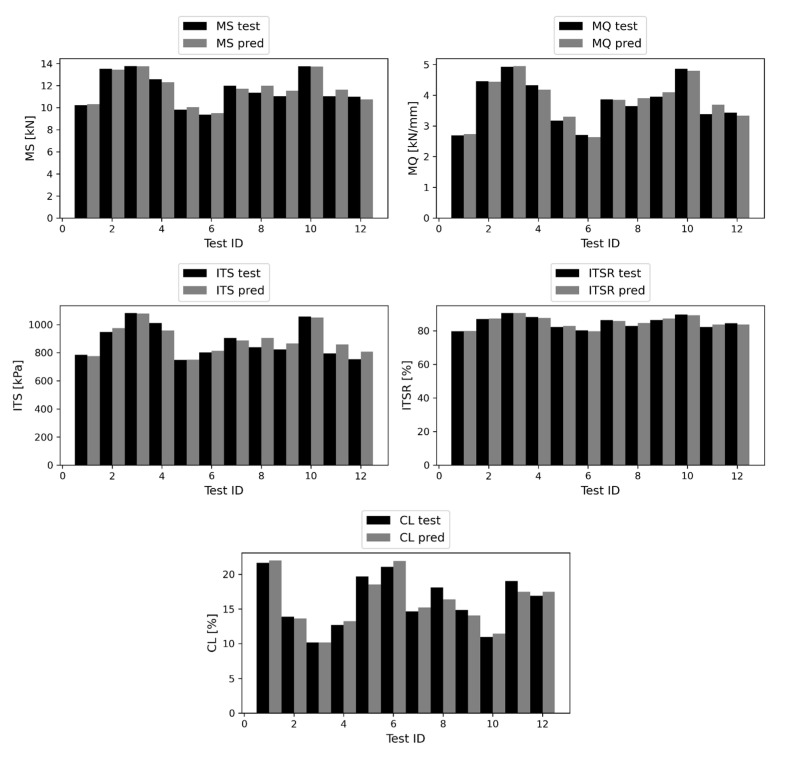
Test vectors and CatBoost predictions of MS (up-left), MQ (up-right), ITS (middle-left), ITSR (middle-right), CL (down).

To provide a deeper insight into the model’s performance, direct comparisons between test vector observations and the corresponding CatBoost predictions are shown in Figure 15. The former are represented by black histograms, and the latter by gray histograms. The ID of each test observation is reported on the horizontal axis. Although relevant fluctuations can be observed in values of each target variable, the differences between the histograms corresponding to the true values and the ones corresponding to the predicted values are not too pronounced.

**Figure 16:**
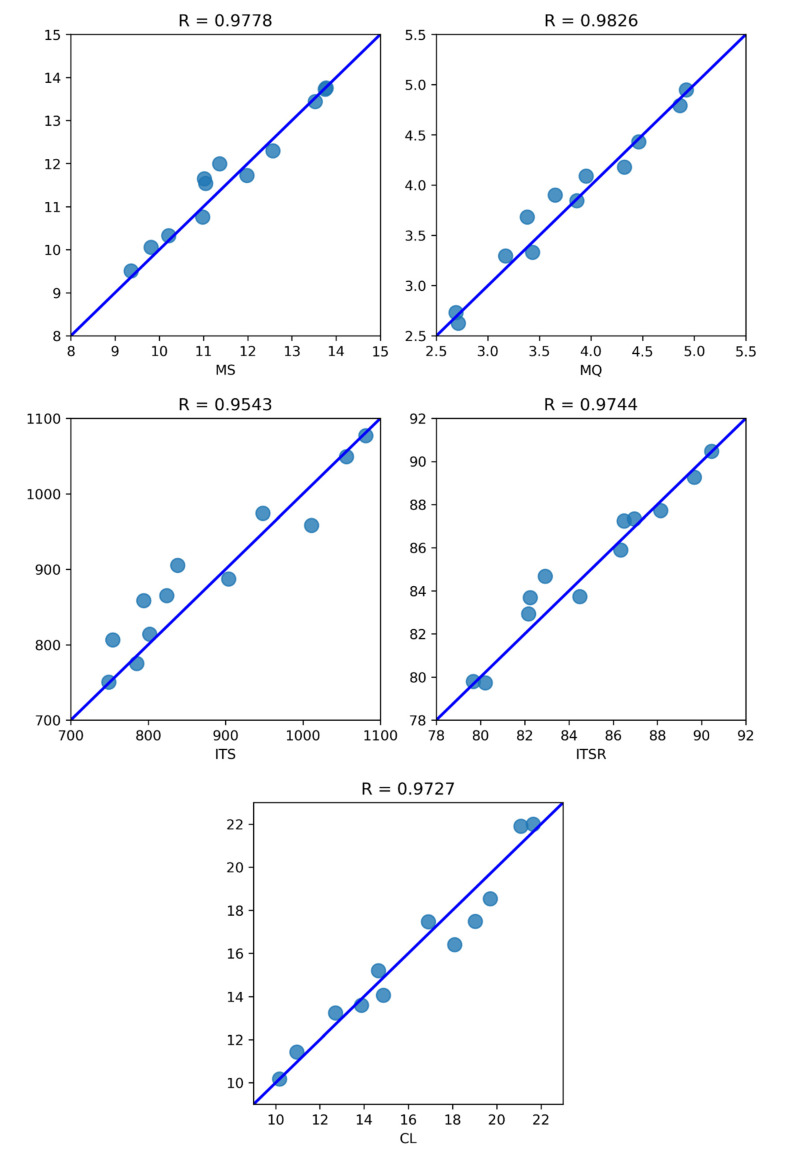
CatBoost model regression plots for MS (**up-left**), MQ (**up-right**), ITS (**middle-left**), ITSR (**middle-right**), CL (**down**).

A further point of view on the accuracy of the predictions made by the CatBoost model was provided by the regression plots represented in Figure 16. The name of the corresponding variable is displayed on the lower edge of each diagram, whereas the Pearson correlation coefficients are reported on the upper edge. CatBoost predictions are represented as light blue circles, whereas a theoretical perfect correspondence between true values and predictions (standing for R = 1) is represented by the line-of-equality displayed in blue. The distance between the predictions and the line-of-equality is very small in all cases, confirming the remarkable results obtained with respect to the Pearson correlation coefficients.

## 4. Conclusions

The present study discusses a laboratory investigation about seven selected alternative fillers that could replace OPC in asphalt concrete mixtures, and a subsequent machine learning model carried out by means of a decision-tree-based CatBoost approach towards mechanical performance prediction. All mixture design parameters were kept constant, except for the type and percentage of fillers, selected from RHA, BD, MD, SD, FA, LD, and SF, and ranging from 4.0% to 8.5% with 1.5% step-size. Marshall, ITS, moisture susceptibility, and Cantabro abrasion loss were carried out for a detailed experimental assessment. The obtained results were implemented into a ML CatBoost methodology, thus allowing MS, MQ, ITS, ITSR, and CL to be simultaneously predicted on the basis of three inputs, namely: a categorical variable distinguishing the filler type, the filler content, and the air voids content. Six different goodness-of-fit metrics were implemented to fully evaluate the prediction reliability, namely MAE, MAPE, MSE, RMSE, R, and R2. Promising results were obtained, with MAPE always lower than 5%, and R and R2 always higher than 0.95 and 0.88, respectively, for each of the five targets. Based on these findings, the following conclusions can be made:

- Based on chemical and physical characterization, all the investigated alternative fillers could potentially be used in asphalt mixture design and replace OPC according to MoRTH standards;
- MoRTH requisites were fully satisfied also in terms of mechanical strength. Except for mixtures prepared with 4.0%, 5.5%, and 7.0% RHA and those prepared with 4.0% MD, all other mixtures showed a MS value above the acceptance threshold of 10 kN. LD and SF at 8.5% even overcame the acceptance requisite for modified bitumen mixtures, with MS values higher than 13 kN;
- All the investigated mixtures also satisfied MoRTH prescriptions in terms of moisture susceptibility, as they provided ITSR values consistently higher than 75%;
- In general terms, LD and SF were found to be the best alternative fillers in asphalt concrete mixtures among those investigated. They not only met MoRTH standards but even provided a better performance than OPC in terms of MS, ITS, ITSR, and CL;
- The data augmentation technique was worthwhile. Remarkable results in terms of all the evaluation metrics were obtained even on the basis of a small-size starting dataset;
- The CatBoost approach resulted in a successful predictive tool that can provide reliable mechanical performance predictions, thus avoiding expensive and time-consuming experimental procedures;
- The entire methodology was developed using Python 3.8.5. It is easily interpretable and implementable by other researchers. Any further application to datasets different from the one analyzed in this study requires new calibration and optimization of model hyperparameters.

The entire experimental investigation was carried out at a laboratory scale. An interesting future development would be to increase the available dataset size, obtained by varying mixtures’ bitumen content or other design parameters. Furthermore, additional properties related to mixtures’ performance could be included, such as rutting resistance or fatigue life. Finally, it would be interesting to perform a similar investigation moving to a full-size scale to understand the field mechanical behavior exhibited by mixtures prepared with the investigated alternative fillers.

## Figures and Tables

**Table 1:** Aggregate properties.

Test Parameter	Method	Results	MoRTH Limits
Cleanliness (dust) (%)	IS 2386 Part I	3	5 (Max)
Bulk specific gravity (g/cm^3^)	IS 2386 Part III	2.68	2–3
Percent wear by Los Angeles abrasion (%)	IS 2386 Part IV	10.6	35 (Max)
Soundness loss by sodium sulphate solution (%)	IS 2386 Part V	3.4	12 (Max)
Soundness loss by magnesium sulphate solution (%)	IS 2386 Part V	3.7	18 (Max)
Flakiness and elongation index (%)	IS 2386 Part I		35 (Max)
- 20 mm		27.93	
- 10 mm		32.13	
Impact strength (%)	IS 2386 Part IV		27 (Max)
- 20 mm		4.15	
- 10 mm		5.91	
Water absorption (%)	IS 2386 Part III	1.67	2 (Max)

**Table 2:** Bitumen properties.

Test Parameter	Method	Results	MoRTH Limits
Absolute viscosity @60 °C (Poises)	IS 1206 (P–2)	2855	2400–3600
Kinematic viscosity @135 °C (cSt)	IS 1206 (P–3)	392	350 (Min)
Flash point Cleveland open cup (°C)	IS 1448 (P–69)	304	250 (Min)
Penetration @25 °C, 100 g, 5 s, (1/10 mm)	IS 1203	49	45 (Min)
Softening point (R&B) (°C)	IS 1205	48	47 (Min)
Matter soluble in trichloroethylene (% by mass)	IS 1216	99.45	99 (Min)
Viscosity ratio @60 °C	IS 1206 (P–2)	1.3	4.0 (Max)
Ductility @25 °C (cm after TFOT)	IS 1208	75	40 (Min)
Specific gravity (g/cm^3^)	IS 1202	0.987	0.97–1.02

**Table 3:** Properties of the investigated mineral fillers.

Test Parameter	Mineral Filler Type							
	RHA	BD	MD	SD	FA	OPC	LD	SF
Specific gravity (g/cm^3^)	2.02	2.56	2.69	2.69	2.32	3.04	2.65	2.20
MBV (g/kg)	4.72	6.25	4.45	3.67	3.86	3.00	3.75	3.85
German filler (g)	65	40	70	85	75	85	97	94
FM	3.21	5.17	2.12	5.38	3.77	4.96	3.03	1.96
Surface area (m^2^/g)	2.31	2.69	4.37	2.70	2.19	1.75	2.70	16.45
pH	10.86	8.67	8.50	12.57	7.30	12.90	10.22	6.98
SiO_2_ (%)	89.67	39.55	0.60	82.37	48.24	21.43	0.48	93.5
CaO (%)	1.88	12.88	55.60	2.79	13.40	66.58	96.57	0.89
Al_2_O_3_ (%)	1.62	15.71	0.40	8.23	24.15	3.01	0.41	0.08
MgO (%)	0.97	3.29	0.10	1.47	1.46	1.39	0.46	0.82
Fe_2_O_3_ (%)	1.06	14.05	0.20	5.27	6.48	4.68	0.32	0.50
Particle shape	Honeycombed	Subangular particles	Subangular particles	Angular particles	Rounded	Granular/ subangular particles	Granular particles	Spherically shaped

**Table 4:** Grid search summary.

Feature	Grid	Selected Value
Number of iterations	100, 250, 500, 1000, 2000	1000
Max depth	1, 2, 3, 4, 5	3
Learning rate	0.5, 0.1, 0.05, 0.01, 0.005	0.1
k-fold Cross-validation	–	4
Overfitting detector	–	20
Loss function	–	*MultiRMSE*

**Table 5:** Performance metrics.

Metric	Definition	Analytical Expression
MAE	Mean absolute error	$\frac{1}{N}\overset{N}{\underset{i=1}{\sum }}\left|y_{T_{i}}-y_{P_{i}}\right|$
MAPE	Mean absolute percentage error	$\frac{1}{N}\overset{N}{\underset{i=1}{\sum }}\left|\frac{y_{T_{i}}-y_{P_{i}}}{y_{T_{i}}}\right|\times 100$
MSE	Mean squared error	$\frac{1}{N}\overset{N}{\underset{i=1}{\sum }}{\left(y_{T_{i}}-y_{P_{i}}\right)}^{2}$
RMSE	Root mean squared error	$\sqrt{\frac{1}{N}\overset{N}{\underset{i=1}{\sum }}{\left(y_{T_{i}}-y_{P_{i}}\right)}^{2}}$
R	Pearson correlation coefficient	$\frac{1}{N-1}\overset{N}{\underset{i=1}{\sum }}\left(\frac{y_{T_{i}}-\mu_{y_{T_{i}}}}{\sigma_{y_{T_{i}}}}\right)\left(\frac{y_{P_{i}}-\mu_{y_{P_{i}}}}{\sigma_{y_{P_{i}}}}\right)$
R^2^	Coefficient of determination	$1-\frac{\sum_{i=1}^{N}{\left(y_{T_{i}}-y_{P_{i}}\right)}^{2}}{\sum_{i=1}^{N}{\left(y_{T_{i}}-\mu_{y_{T_{i}}}\right)}^{2}}$

**Table 6:** Statistical description of CatBoost model variables.

Variable	Description	U.M.	Count	Mean	Std. Dev.
CV	Categorical variable–filler type	–	56	–	–
Fc	Filler content	%	56	6.25	1.51
AV	Air voids	%	56	4.42	0.40
MS	Marshall stability	kN	56	11.88	1.42
MQ	Marshall quotient	kN/mm	56	3.93	0.74
ITS	Indirect tensile strength	kPa	56	901.61	107.82
ITSR	Indirect tensile strength ratio	%	56	85.35	3.76
CL	Cantabro Loss	%	56	15.73	4.05

**Table 7:** CatBoost model performance metrics.

Metric	MS	MQ	ITS	ITSR	CL
MAE	0.2595	0.1099	29.5495	0.6441	0.7317
MAPE	2.3328	3.0829	3.4862	0.7669	4.3340
MSE	0.1101	0.0195	1432.5907	0.6495	0.7670
RMSE	0.3319	0.1396	37.8496	0.8059	0.8758
R	0.9778	0.9826	0.9543	0.9744	0.9727
R^2^	0.9478	0.9626	0.8885	0.9446	0.9437

## Data Availability

The data that support the findings of this study are available from the corresponding author upon reasonable request.

## Abbreviations

AM	Asphalt Mixture
AV	Air Voids
BD	Brick Dust
BF	Backhouse filler
CL	Cantabro Loss
CV	Categorical Variable
FA	Fly Ash
Fc	Filler content
FM	Fineness Modulus
ITS	Indirect Tensile Strength
ITSR	Indirect Tensile Strength Ratio
MBV	Methylene blue value
MD	Marble Dust
MF	Marshall Flow
ML	Machine Learning
MQ	Marshall Quotient
MS	Marshall Stability
LD	Limestone Dust
OPC	Ordinary Portland Cement
QD	Quarry Dust
RHA	Rice Husk Ash
SD	Stone Dust
SF	Silica Fume
VMA	Voids in the Mineral Aggregate
VFB	Voids Filled with Bitumen

## References

[1] Wang L., Gong H., Hou Y., Shu X., Huang B. (2017). Advances in pavement materials, design, characterisation, and simulation. Road Mater. Pavement Des..

[2] Yilmaz M., Kök B.V., Kulolu N. (2011). Effects of using asphaltite as filler on mechanical properties of hot mix asphalt. Constr. Build. Mater..

[3] Chen M., Lin J., Wu S., Liu C. (2011). Utilization of recycled brick powder as alternative filler in asphalt mixture. Constr. Build. Mater..

[4] Veytskin Y., Bobko C., Castorena C., Kim Y.R. (2015). Nanoindentation investigation of asphalt binder and mastic cohesion. Constr. Build. Mater..

[5] Dash S.S., Panda M. (2018). Influence of mix parameters on design of cold bituminous mix. Constr. Build. Mater..

[6] Miró R., Martínez A.H., Pérez-Jiménez F.E., Botella R., Álvarez A. (2017). Effect of filler nature and content on the bituminous mastic behaviour under cyclic loads. Constr. Build. Mater..

[7] Raposeiras A.C., Movilla-Quesada D., Bilbao-Novoa R., Cifuentes C., Ferrer-Norambuena G., Castro-Fresno D. (2018). The use of copper slags as an aggregate replacement in asphalt mixes with RAP: Physical—chemical and mechanical behavioural analysis. Constr. Build. Mater..

[8] Xue Y., Hou H., Zhu S., Zha J. (2009). Utilization of municipal solid waste incineration ash in stone mastic asphalt mixture: Pavement performance and environmental impact. Constr. Build. Mater..

[9] Ogundipe O.M. (2016). Marshall Stability and Flow of Lime-modified Asphalt Concrete. Transp. Res. Procedia.

[10] Lesueur D., Petit J., Ritter H.J. (2013). The mechanisms of hydrated lime modification of asphalt mixtures: A state-of-the-art review. Road Mater. Pavement Des..

[11] Rashwan N.K. (2016). Hot Mix Asphalt (HMA) Performance as Affected by Limestone Powder Filler Content. World Appl. Sci. J..

[12] Chrismer J.L., Durham S.A. High Volume Fly Ash Concrete for Highway Pavements. Proceedings of the Green Streets and Highways Conference.

[13] Abdel-Wahed T.A., Rashwan N.K. Application of Cement Dust and OPC as Mineral Filler in the binder Hot Mix Asphalt. Proceedings of the 5th Annual International Conference on Asphalt, Pavement Engineering and Infrastructure.

[14] Mazzoni G., Virgili A., Canestrari F. (2019). Influence of different fillers and SBS modified bituminous blends on fatigue, self-healing and thixotropic performance of mastics. Road Mater. Pavement Des..

[15] Schneider M., Romer M., Tschudin M., Bolio H. (2011). Sustainable cement production-present and future. Cem. Concr. Res..

[16] Wang H., Al-Qadi I.L., Faheem A.F., Bahia H.U., Yang S.H., Reinke G.H. (2011). Effect of Mineral Filler Characteristics on Asphalt Mastic and Mixture Rutting Potential. Transp. Res. Rec..

[17] Tiwari N., Satyam N. (2021). Evaluation of Strength and Water Susceptibility Performance of Polypropylene Fiber-Reinforced and Silica Fume-Modified Hot Mix Asphalt. Adv. Civ. Eng. Mater..

[18] Modarres A., Alinia Bengar P. (2019). Investigating the Indirect Tensile Stiffness, Toughness and Fatigue Life of Hot Mix Asphalt Containing Copper Slag Powder. Int. J. Pavement Eng..

[19] Choudhary J., Kumar B., Gupta A. (2019). Performance Evaluation of Bauxite Residue Modified Asphalt Concrete Mixes. Eur. J. Environ. Civ. Eng..

[20] Lukjan A., Iyaruk A., Somboon C. (2022). Evaluation on Mechanical Deterioration of the Asphalt Mixtures Containing Waste Materials when Exposed to Corrosion Solutions. Int. J. Eng. Technol..

[21] Tessema A.T., Wolelaw N.M., Alene G.A. (2022). Experimental Evaluation of Coffee Husk Ash as a Filler in Hot Mix Asphalt Concrete Productions. Adv. Civ. Eng..

[22] Awed A.M., Tarbay E.W., El-Badawy S.M., Azam A.M. (2022). Performance characteristics of asphalt mixtures with industrial waste/by-product materials as mineral fillers under static and cyclic loading. Road Mater. Pavement Des..

[23] Hamedi G.H., Esmaeeli M.R., Najafi Moghaddam Gilani V., Hosseinian S.M. (2021). The effect of aggregate-forming minerals on thermodynamic parameters using surface free energy concept and its relationship with the moisture susceptibility of asphalt mixtures. Adv. Civ. Eng..

[24] Sholevar N., Golroo A., Esfahani S.R. (2022). Machine learning techniques for pavement condition evaluation. Autom. Constr..

[25] Hou Y., Li Q., Zhang C., Lu G., Ye Z., Chen Y., Wang L., Cao D. (2021). The state-of-the-art review on applications of intrusive sensing, image processing techniques, and machine learning methods in pavement monitoring and analysis. Engineering.

[26] Pasetto M., Baldo N. (2016). Numerical visco-elastoplastic constitutive modelization of creep recovery tests on hot mix asphalt. J. Traffic Transp. Eng..

[27] De Oliveira Junior M., De Farias M.M. (2020). A simple numerical methodology to simulate creep and recovery tests in HMA. Constr. Build. Mater..

[28] Cao P., Leng Z., Shi F., Zhou C., Tan Z., Wang Z. (2020). A novel visco-elastic damage model for asphalt concrete and its numerical implementation. Constr. Build. Mater..

[29] Sun B., Hao P., Zhang H., Liu J. (2022). Establishment and verification of a developed viscoelastic damage model and creep instability criterion for modified fine asphalt mortar. Mater. Struct..

[30] Pasetto M., Baldo N. (2015). Computational analysis of the creep behaviour of bituminous mixtures. Constr. Build. Mater..

[31] Baldo N., Miani M., Rondinella F., Valentin J., Vackcová P., Manthos E. (2022). Stiffness Data of High-Modulus Asphalt Concretes for Road Pavements: Predictive Modeling by Machine-Learning. Coatings.

[32] Baldo N., Miani M., Rondinella F., Manthos E., Valentin J. (2022). Road Pavement Asphalt Concretes for Thin Wearing Layers: A Machine Learning Approach towards Stiffness Modulus and Volumetric Properties Prediction. Period. Polytech. Civ. Eng..

[33] Upadhya A., Thakur M.S., Sihag P., Kumar R., Kumar S., Afeeza A., Afzal A., Saleel C.A. (2022). Modelling and prediction of binder content using latest intelligent machine learning algorithms in carbon fiber reinforced asphalt concrete. Alex. Eng. J..

[34] Behbahani H., Hamedi G.H., Gilani V.N.M. (2020). Predictive model of modified asphalt mixtures with nano hydrated lime to increase resistance to moisture and fatigue damages by the use of deicing agents. Constr. Build. Mater..

[35] Gilani V.N.M., Hosseinian S.M., Behbahani H., Hamedi G.H. (2020). Prediction and pareto-based multi-objective optimization of moisture and fatigue damages of asphalt mixtures modified with nano hydrated lime. Constr. Build. Mater..

[36] Marcelino P., De Lurdes Antunes M., Fortunato E., Gomes M.C. (2021). Machine learning approach for pavement performance prediction. Int. J. Pavement Eng..

[37] Zeiada W., Dabous S.A., Hamad K., Al-Ruzouq R., Khalil M.A. (2020). Machine learning for pavement performance modelling in warm climate regions. Arab. J. Sci. Eng..

[38] Kandhal P.S., Lynn C.Y., Parker F. (1998). Characterization Tests for Mineral Fillers Related to Performance of Asphalt Paving Mixtures. Transp. Res. Rec. J. Transp. Res. Board.

[39] Sousa J.B., Way G., Harvey J.T., Hines M. (1995). Comparison of mix design concepts. Transp. Res. Rec..

[40] Pasandín A.R., Pérez I. (2015). Overview of bituminous mixtures made with recycled concrete aggregates. Constr. Build. Mater..

[41] Al-Ammari M.A.S., Jakarni F.M., Muniandy R., Hassim S. (2019). The effect of aggregate and compaction method on the physical properties of hot mix asphalt. IOP Conf. Ser. Mater. Sci. Eng..

[42] Ozgan E. (2011). Artificial neural network based modelling of the Marshall stability of asphalt concrete. Expert Syst. Appl..

[43] Zavrtanik N., Prosen J., Tušar M., Turk G. (2016). The use of artificial neural networks for modeling air void content in aggregate mixture. Autom. Constr..

[44] (1995). Superpave Level 1 Mix Design, Superpave Series No. 2 (SP-2).

[45] (2015). Standard Test Method for Marshall Stability and Flow of Asphalt Mixtures.

[46] Zoorob S.E., Suparma L.B. (2000). Laboratory Design and Investigation of the Properties of Continuously Graded Asphaltic Concrete Containing Recycled Plastics Aggregate Replacement (Plastiphalt). Cem. Concr. Compos..

[47] (2017). Standard Test Method for Indirect Tensile (IDT) Strength of Asphalt Mixtures.

[48] (2019). TxDOT: Tex-245-F Test Procedure for Cantabro Loss.

[49] Prokhorenkova L., Gusev G., Vorobev A., Dorogush A.V., Gulin A. (2017). Catboost: Unbiased boosting with categorical features. arXiv.

[50] Lou Y., Obukhov M. Bdt: Gradient boosted decision tables for high accuracy and scoring efficiency. Proceedings of the 23rd ACM SIGKDD International Conference on Knowledge Discovery and Data Mining (KDD’17).

[51] Bentéjac C., Csörgő A., Martínez-Muñoz G. (2021). A comparative analysis of gradient boosting algorithms. Artif. Intell. Rev..

[52] Chen T., Guestrin C. Xgboost: A scalable tree boosting system. Proceedings of the 22nd ACM SIGKDD International Conference on Knowledge Discovery and Data Mining (KDD’16).

[53] Ke G., Meng Q., Finley T., Wang T., Chen W., Ma W., Ye Q., Liu T.Y. Lightgbm: A highly efficient gradient boosting decision tree. Proceedings of the 31st International Conference on Neural Information Processing Systems (NIPS’17).

[54] Baldo N., Miani M., Rondinella F., Celauro C. (2021). A Machine Learning Approach to Determine Airport Asphalt Concrete Layer Moduli Using Heavy Weight Deflectometer Data. Sustainability.

[55] James G., Witten D., Hastie T., Tibshirani R. (2013). An Introduction to Statistical Learning with Applications in R.

[56] Rahaman M.S., Liono J., Ren Y., Chan J., Kudo S., Rawling T., Salim F.D. (2020). An ambient-physical system to infer concentration in open-plan workplace. IEEE Internet Things J..

[57] Oh C., Han S., Jeong J. (2020). Time-Series Data Augmentation Based on Interpolation. Procedia Comput, Sci..

[58] Akima H. (1974). A method of bivariate interpolation and smooth surface fitting based on local procedures. Commun. ACM.

[59] Akima H. (1970). A new method of interpolation and smooth curve fitting based on local procedures. J. ACM.

[60] Ministry of Road Transport and Highways (MoRTH) (2013). Specifications for Road and Bridges Works, 5th Revision.

[61] Kutuk-Sert T. (2016). Stability Analyses of Submicron-Boron Mineral Prepared by Mechanical Milling Process in Concrete Roads. Constr. Build. Mater..

[62] Nikolaides A. (2015). Highway Engineering—Pavements, Materials and Control of Quality.

[63] Akbulut H., Gürer C., Çetin S., Elmaci A. (2012). Investigation of using granite sludge as filler in bituminous hot mixtures. Constr. Build. Mater..

[64] Huang B., Shu X., Chen X. (2007). Effects of mineral fillers on hot-mix asphalt laboratory-measured properties. Int. J. Pavement Eng..

[65] Arabani M., Tahami S.A., Taghipoor M. (2017). Laboratory investigation of hot mix asphalt containing waste materials. Road Mater. Pavement Des..

[66] Kuity A., Jayaprakasan S., Das A. (2014). Laboratory Investigation on Volume Proportioning Scheme of Mineral Fillers in Asphalt Mixture. Constr. Build. Mater..

[67] Pallant J. (2016). SPSS Survival Manual: A Step by Step Guide to Data Analysis Using IBM SPSS.

[68] Saleh R., Fleyeh H. (2022). Using supervised machine learning to predict the status of road signs. Transp. Res. Proc..

